# Histopathologic evidence of VEGF in early neovascular AMD: from a 1992 hypothesis to a 1994 discovery — a historical perspective

**DOI:** 10.1186/s40942-025-00779-x

**Published:** 2025-12-13

**Authors:** K. Alexander Dastgheib

**Affiliations:** Dastgheib Vision and Innovation Eye Institute, Newport Beach, California USA

**Keywords:** Vascular endothelial growth factor (VEGF), Age-related macular degeneration (AMD), Neovascular age-related macular degeneration (nAMD), Choroidal neovascularization (CNV), Macular neovascularization (MNV), Retinal pigment epithelium (RPE), Immunohistochemistry, Anti-VEGF therapy, Hypoxia, Histopathology

## Abstract

**Background:**

In the early1990s, neovascular age-related macular degeneration (nAMD) was the leading cause of irreversible vision loss in older adults, yet its molecular basis remained unknown. In 1992, a hypothesis was proposed in which localized hypoxia could trigger vascular endothelial growth factor (VEGF)-mediated choroidal neovascularization in nAMD. Although hypoxia was recognized in ischemic retinopathies, nAMD was not considered a hypoxia-mediated retinal vascular disease.

**Main body:**

In 1994, this hypothesis was tested using antigen retrieval immunohistochemistry on paraffin-embedded whole human eye sections with early nAMD. The study demonstrated strong VEGF immunoreactivity in the retinal pigment epithelium in the macular area but not in normal control eyes, providing the first direct histopathologic evidence of VEGF expression at the site of disease in intact human eyes with early nAMD. Until that point, the role of VEGF in ischemic retinopathies was being uncovered, but its involvement in early-stage nAMD had not yet been demonstrated.

**Conclusion:**

The 1992–1994 work established both the hypothesis and the first direct tissue evidence linking VEGF to early nAMD. This discovery, made just over a decade before the advent of anti-VEGF therapy, anticipated one of ophthalmology’s most transformative achievements, preserving vision for millions worldwide.

## Background: genesis of a hypothesis

In the early 1990s, neovascular age-related macular degeneration (nAMD) was the leading cause of irreversible blindness in older adults in developed countries [1]. Its defining feature, choroidal neovascularization, was well-documented by fluorescein angiography and histopathology, yet its molecular underpinnings remained poorly understood. During that era, the only available treatment was thermal laser photocoagulation, [2] which was often destructive and rarely helpful. For many patients, clinicians could offer little beyond observation.

By that time, several eye pathology reports had detailed inflammatory findings in AMD [3–5]. In a 1992 case report of nAMD, later published in 1994, titled “Granulomatous Reaction to Bruch’s Membrane in Age-Related Macular Degeneration”[6] multinucleated giant cells were identified in close proximity to Bruch’s membrane raising the possibility that inflammation might contribute to choroidal neovascularization. At that point, specific mediators of the angiogenic response in nAMD remained elusive.

A few years earlier, in 1989, two independent groups identified and cloned the same molecule, which one termed vascular endothelial growth factor (VEGF) and the other vascular permeability factor (VPF), published in back-to-back articles in *Science* [7, 8]. One group characterized it as a secreted mitogen specific for endothelial cells, while the other demonstrated its potent vascular permeability activity. In 1992, VEGF was described as a mediator of hypoxia-induced angiogenesis in glioma cells [9]. A connection to nAMD was made immediately: [6] in AMD, the normally 4 μm-thick Bruch’s membrane in the macula [10] can become significantly thickened by basal linear deposits, which accumulate external to the basement membrane of the retinal pigment epithelium (RPE) within the inner collagenous zone of Bruch’s membrane [11]. It was hypothesized that this thickening of the inner aspect of Bruch’s membrane, which at times separates from the remainder of Bruch’s membrane, could act as a barrier to oxygen diffusion from the choriocapillaris [6]. Since the outer third of the retina receives its oxygen supply from the choroidal capillaries, [12] while the inner two-thirds are nourished by the retinal vasculature, such a barrier could induce chronic hypoxia in the outer retina, potentially triggering VEGF-driven choroidal neovascularization [6]—a concept then novel and counterintuitive. At that period, hypoxia was recognized in ischemic retinal disorders such as diabetic retinopathy and retinal vascular occlusion, but choroidal neovascularization in AMD was not yet viewed as a hypoxia-mediated retinal or choroidal vascular disorder.

## Main text

### The 1994 discovery: whole-eye evidence of VEGF expression in early nAMD

**Fig. 1:**
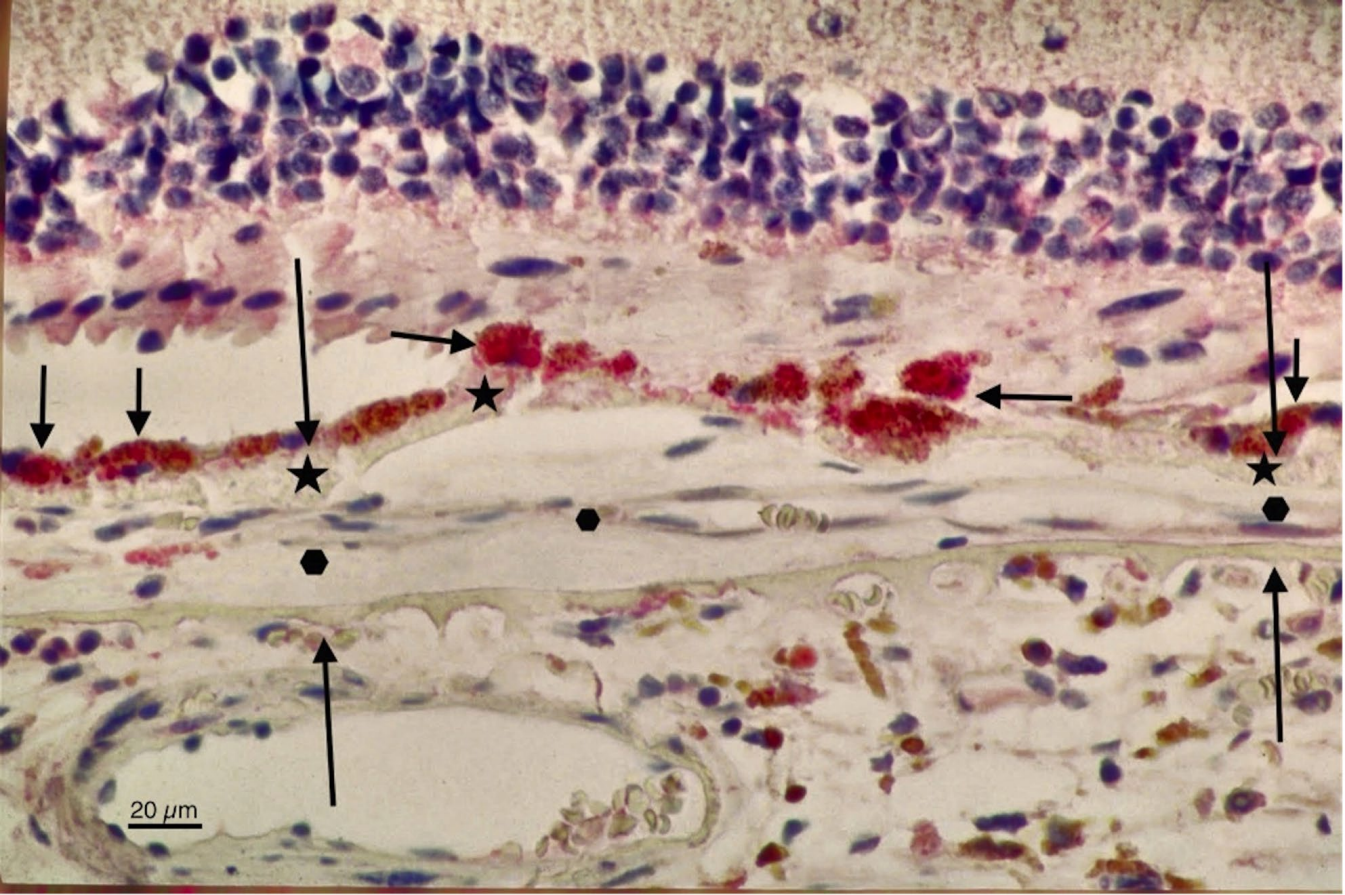
Immunohistochemical localization of VEGF in early-stage nAMD (1994). Strong red chromogen (Fast Red substrate-chromogen system) highlights VEGF expression (short arrows) in the retinal pigment epithelium overlying Bruch’s membrane (between long arrows) in the macular area. The inner aspect of Bruch’s membrane is thickened (stars) and separated from its outer portion by early, thin choroidal neovascularization (15–45 μm; hexagons). The image was produced using a monoclonal anti-VEGF antibody on a formalin-fixed, paraffin-embedded section of a whole human eye with early nAMD and counterstained with hematoxylin. A later white-balance correction (contrast-limited adaptive histogram equalization [CLAHE] in L*a*b* color space) was applied to neutralize the background and enhance contrast. The faint pink background tint represents a non-specific effect that occurs in sections with strong chromogen deposition and thus reflects the presence of VEGF-positive staining elsewhere in the tissue. No changes were made to tissue, staining, or overlaid annotations. Original magnification x 450

**Fig. 2:**
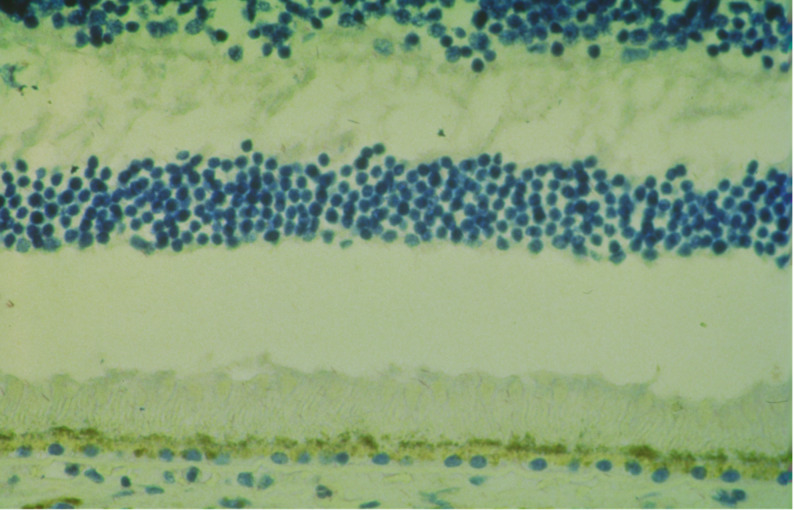
Immunohistochemistry for VEGF in the macular area of a normal human eye (1994). Original photomicrograph. Normal control eye processed with the same methodology and red chromogen (Fast Red substrate-chromogen system) as in Fig. 1 shows no VEGF immunoreactivity in the retinal pigment epithelium or other macular structures. The absence of chromogen deposition yields only hematoxylin-stained nuclei against a lighter yellowish-white background, in contrast to the faint pink background tint observed in positive macular sections (Figs. 1). Original magnification x 450

In 1994, the opportunity arose to test this hypothesis directly using antigen retrieval by microwave heating, a technique then recently introduced in general histopathology for paraffin-embedded tissue [13] but rarely applied to ocular specimens. Immunohistochemistry for VEGF was performed on 6 μm paraffin-embedded whole human eye sections from donors with early nAMD. This adaptation overcame the limitations of frozen-section methods, which were unsuitable for intact human globes. The results were striking: strong VEGF immunoreactivity in the RPE in the macular area in eyes with early nAMD (Fig. 1), [14, 15] with no signal in normal control eyes (Fig. 2). These findings provided the first direct histopathologic evidence of VEGF at the site of disease in whole human eyes with early nAMD.

### Context and contemporary investigations

The discovery titled “Vascular Endothelial Growth Factor (VEGF) in Neovascular Age-related Macular Degeneration” was presented in April 1995 at the Wilmer Residents Association meeting at Johns Hopkins University, [14] and a month later at Association for Research in Vision and Ophthalmology (ARVO). The work was published in the *Investigative Ophthalmology & Visual Science* (*IOVS*), ARVO supplement, 1995 [15]. It represented an early bridge between molecular angiogenesis research and macular pathology in nAMD.

Meanwhile, other groups were uncovering the role of VEGF in ischemic retinal diseases, which helped establish a mechanistic framework for ocular angiogenesis. VEGF was detected in ocular fluids of patients with ischemic retinal diseases such as active proliferative diabetic retinopathy and retinal vein occlusion, whereas nAMD samples were negative for VEGF [16]. In primate models, retinal ischemia induced by laser occlusion of all branch retinal veins produced synchronous and proportional rises in aqueous VEGF/VPF levels associated with severity of iris neovascularization [17]. VEGF inhibition prevented these lesions [18]. Additional studies showed that soluble VEGF receptors reduced neovascularization in a murine model of retinopathy of prematurity [19]. Collectively, these ischemic retinopathy studies confirmed that VEGF could drive angiogenesis under hypoxic stress, but no direct evidence yet linked VEGF to nAMD, the most prevalent cause of central vision loss. Reflecting that uncertainty, Ferrara concluded in his June 1995 *Laboratory Investigation* editorial, ”Whether VEGF plays a role in the pathogenesis of [age-related macular degeneration] is an attractive possibility that deserves to be explored.” [20] By the time of that writing, the exploration Ferrara anticipated had in fact already been completed and reported [14, 15]. 

The absence of detectable VEGF in early ocular fluid studies of nAMD [16] reflected the limited sensitivity of radio-immunometric assays then in use, which had a detection threshold of approximately 50 pg/mL. Many years later, ELISA-based methods, capable of detecting VEGF at levels as low as 3.5 pg/mL, identified measurable aqueous VEGF across all morphologic forms of macular neovascularization (MNV) secondary to nAMD, each representing clinically active, exudative disease with OCT-confirmed increases in central macular thickness [21]. In that study, type 3 MNV showed higher VEGF levels than types 1 and 2, and the prominent cystoid intraretinal fluid characteristic of type 3 MNV—recently highlighted through advanced AI-based imaging analysis [22]—aligns with these findings. Together, these observations, emerging nearly two to three decades later, reinforce the 1994 whole-eye evidence, which demonstrated VEGF expression directly within intact human macular tissue at a stage when fluid-phase VEGF may remain below even modern assay detection limits because of compartmentalization and the absence of exudation in the earliest form of disease.

While ischemic retinopathies provided mechanistic context, the 1994 whole-eye investigation provided direct histopathologic proof of strong VEGF expression in early-stage nAMD, [14, 15] a classically non-ischemic form of ocular neovascularization. By localizing VEGF to the RPE in situ, this study established the previously missing link between the hypothesized hypoxia-driven mechanism [6] and tissue disease in early nAMD. As later noted by Miller in her 2014 Champalimaud Vision Award lecture, “in 1996…we had extensive evidence that VEGF had a key role in ischemic retinal disease in animal models and in humans. For neovascular AMD, there was perhaps less direct evidence for a causal role of VEGF, but there was definitely an unmet need...” [23]. The 1994 demonstration [14, 15] helped close that knowledge gap, showing that VEGF expression was not confined to ischemic retinopathies but also present in the earliest stages of neovascularization in AMD.

Among three contemporaneous investigations focused specifically on VEGF in nAMD, two employed surgically excised choroidal neovascular membranes, [24, 25] whereas the 1994 study—presented at Hopkins and later at ARVO—examined intact whole-eye sections [14, 15]. The excised membrane studies confirmed VEGF presence but primarily in fibrotic, late-stage lesions, where VEGF localized to transdifferentiated RPE or fibroblast-like cells, consistent with an epithelial-mesenchymal-transition response. The thickness of the excised tissue itself—hundreds of micrometers—contrasted with early choroidal neovascularization measuring as little as 15 μm in whole-eye sections (Fig. 1), further reflecting their advanced stage. By preserving native macular architecture and the molecular-spatial context of the tissue, the whole-eye study revealed VEGF expression in morphologically normal, non-transdifferentiated RPE at the earliest angiogenic stages.

Together, these findings delineated a temporal and phenotypic progression: VEGF was upregulated in normal RPE cells during early disease, initiating angiogenesis, and later shifting to transdifferentiated RPE and fibroblast-like cells as fibrosis and scarring developed. This sequence suggested that VEGF-driven choroidal neovascularization preceded structural degeneration or scarring, reinforcing the importance of early detection and intervention. By demonstrating VEGF expression in early-stage, non-excised human tissue, [14, 15] this evidence helped address a key uncertainty in the biological rationale for targeting VEGF in nAMD.

### From pathologic insight to therapeutic impact

At a time when no molecular target for nAMD therapy had been identified, the 1992–1994 work provided both a biologic hypothesis and direct histopathologic evidence of VEGF expression within *early* nAMD tissue [6, 14, 15]. Though not alone sufficient to establish causality, these findings offered the first tissue-based rationale in early-stage disease that anticipated later therapeutic developments, with the causal role of VEGF subsequently confirmed in clinical trials of anti-VEGF agents. In retrospect, once VEGF had been identified as the therapeutic target in nAMD, the conceptual leap to pharmacologic inhibition was straightforward. Pharmaceutical efforts soon focused on optimizing molecular size, retinal penetration, and dosing durability rather than redefining the target itself. Clinical experience confirmed that full-length antibodies such as bevacizumab and smaller antibody fragments such as ranibizumab achieved comparable efficacy, [26] differing mainly in formulation and duration of action. Later agents such as aflibercept achieved similar results using a fusion-protein design that bound multiple VEGF isoforms, [27] while earlier agents such as pegaptanib demonstrated proof of concept for VEGF inhibition in clinical use [28]. The pivotal advance lay not in incremental drug design but in the original recognition of VEGF as a key pathogenic factor in nAMD—a realization that ultimately set the stage for targeted biologic therapy in a previously untreatable disease. Viewed collectively, the 1992–1994 investigations bridged molecular hypothesis, pathologic confirmation, and translational impact—linking the earliest biologic insight to the therapeutic revolution that would unfold in the following decade.

## Conclusions

The validation of anti-VEGF therapy in nAMD stands among the most significant achievements in ophthalmology, preserving sight in millions worldwide [29, 30]. Subsequent approvals extended its use to diabetic macular edema [31] and vein occlusion-related macular edema [32, 33]. From a historical perspective, the 1994 whole-eye demonstration of VEGF in early nAMD represented more than a technical accomplishment; it revealed a disease mechanism that would later underpin a global therapeutic paradigm centered on anti-VEGF therapy in nAMD. As with other moments in science where early visual evidence—such as Fig. 1—preceded broad recognition, this demonstration, though publicly presented in 1995, remains under-recognized today. Such images may not immediately alter clinical practice, but their translational significance often becomes evident only in retrospect as collective understanding matures. In this case, the histopathologic image preceded the therapeutic paradigm by over a decade, and its enduring significance continues to grow clearer with ongoing clinical and scientific progress.^1^  

## Data Availability

No datasets were generated or analysed during the current study.

## Footnotes

1 Though in a different domain, the scientific trajectory of *Photograph 51*—the x-ray diffraction image captured by Rosalind Franklin that revealed key features of the DNA double-helix—illustrates how early visual evidence, even when noted at the time, may not be fully appreciated or broadly recognized until later, yet can ultimately precede and shape paradigm shifts.

## Abbreviations

nAMD: Neovascular age-related macular degeneration
VEGF: Vascular endothelial growth factor
VPF: Vascular permeability factor
RPE: Retinal pigment epithelium
CLAHE: Contrast-limited adaptive histogram equalization
L*a*b*: Lightness (L*), the green-red axis (a*), and the blue-yellow axis (b*)
ARVO: Association for Research in Vision and Ophthalmology
IOVS: Investigative Ophthalmology and Visual Science
ELISA: Enzyme-linked immunosorbent assay
MNV: Macular neovascularization
OCT: Optical coherence tomography
AI: Artificial intelligence
DNA: Deoxyribonucleic acid
